# Supplementary material for: Machine learning aided construction of the quorum sensing communication network for human gut microbiota
Source: Nat Commun. 2022 Jun 2;13:3079. doi: 10.1038/s41467-022-30741-6 (PMC9163137; doi:10.1038/s41467-022-30741-6)
Supplement: Supplementary file 1 — Supplementary Information [file 41467_2022_30741_MOESM1_ESM.pdf]

Supplementary Information

Machine Learning Aided Construction of the Quorum Sensing Communication Network for Human Gut Microbiota

Shengbo Wu[1,2], Jie Feng[3], Chunjiang Liu[1,2], Hao Wu[4], Zekai Qiu[1], Jianjun Ge[1], Shuyang Sun[1], Xia Hong[1], Yukun Li[1], Xiaona Wang[1], Aidong Yang[5*], Fei Guo[6*], and Jianjun Qiao[1,4,7*]

[1] School of Chemical Engineering and Technology, Tianjin University, Tianjin, 300072, China

[2] State Key Laboratory of Chemical Engineering, Tianjin University, Tianjin 300072, China

[3] School of Computer Science and Technology, College of Intelligence and Computing, Tianjin University, Tianjin 300350, China

[4] Zhejiang Shaoxing Research Institute of Tianjin University, Shaoxing, 312300, China

[5] Department of Engineering Science, University of Oxford, Oxford OX1 3PJ, UK

[6] School of Computer Science and Engineering, Central South University, Changsha 410083, China

[7] Key Laboratory of Systems Bioengineering, Ministry of Education (Tianjin University), Tianjin, 300072, China

*Correspondence and requests for materials should be addressed to

Aidong Yang (aidong.yang@eng.ox.ac.uk)

Fei Guo (email: guofei@csu.edu.cn)

Jianjun Qiao (email: jianjunq@tju.edu.cn)

1. Structure of the work and key learnings

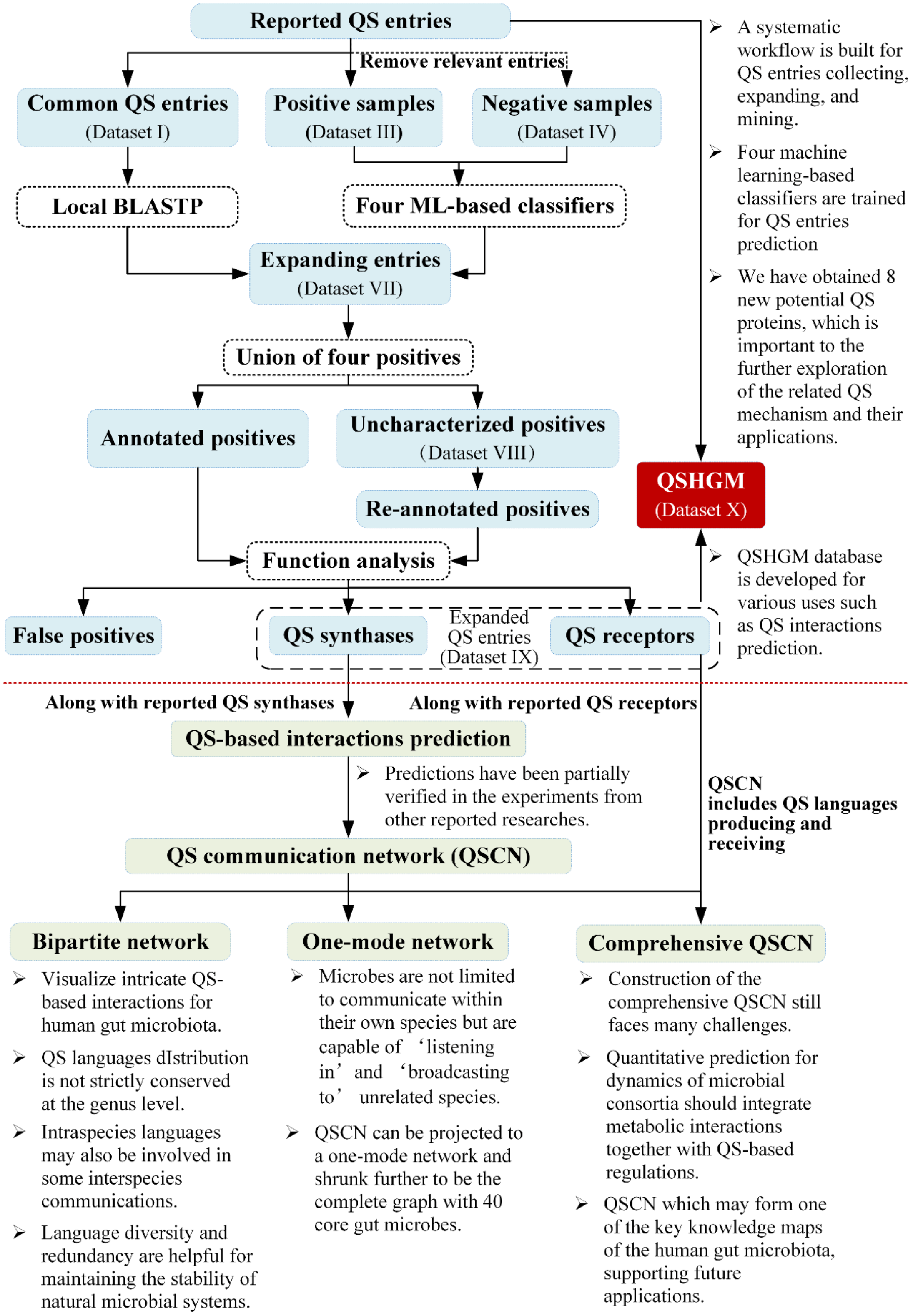

Supplementary Figure 1. A diagram illustrating the structure of the work and key learnings from different parts of the study.

2. Overlaps analysis for the annotated and uncharacterized positives

We want to mine potential QS entries for human gut microbes as inclusively as possible. Different classifiers can help us obtain different positives to mine more potential QS entries. We have manually checked their annotations and divided the union of positives into annotated positives (AP) and uncharacterized positives (UP) (Figure 3B), which were analyzed further for their specific overlaps (Supplementary Figure 2) (see more details in Supplementary Data 12). As illustrated in the red box of Supplementary Figure 2, there are 882 and 34 entries shared only by one classifier for annotated and uncharacterized positives, respectively, which indicates the need for the union of the four positives to cover more potential QS entries. Note that the union of positives from SVM and KNN classifiers are predominant, the positives from RF (65 entries for AP, 7 entries for UP) or DNN (134 entries for AP, 8 entries for UP) classifier can supplement entries to some extent. When the positives of three classifiers are combined together, such as SVM/KNN/RF and SVM/KNN/DNN, the positives from the fourth classifier will contribute even less. This indicates that the positives from the above classifiers can cover most of the entries, which is why we did not use any further classifiers.

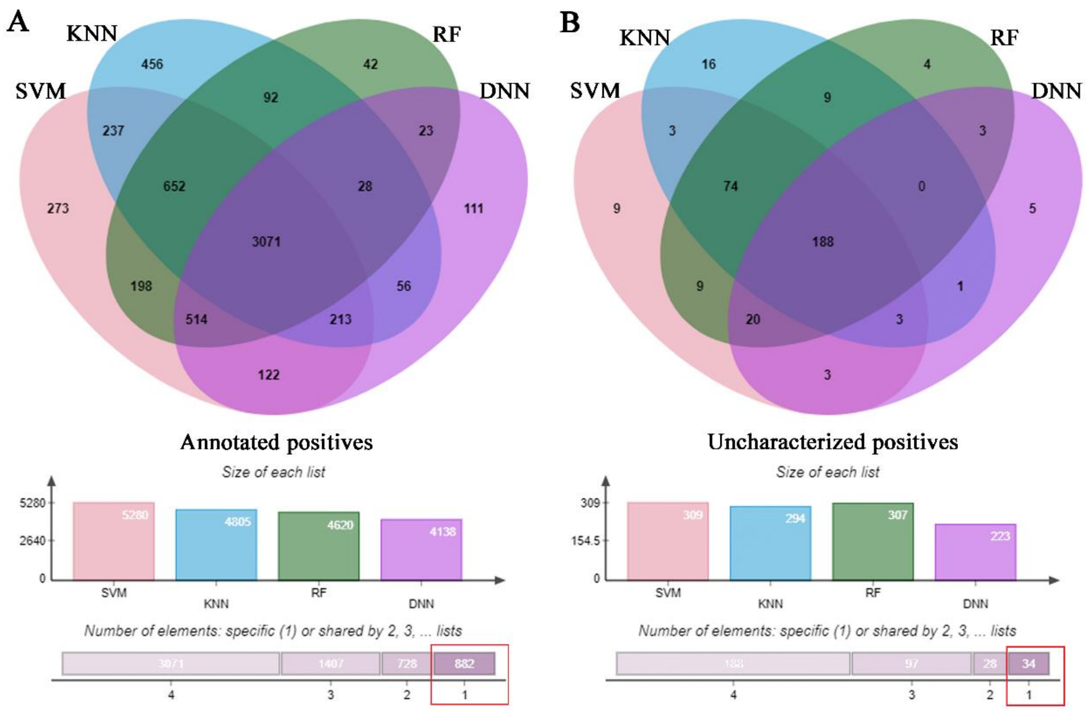

Supplementary Figure 2. The overlaps for the annotated and uncharacterized positives from the four ML-based classifiers.

3. QSHGM browsing and searching

To enable user-friendly browsing and searching for entries identified in this work, we constructed a comprehensive QS-related database of human gut microbiota (QSHGM), which is freely available at: http://www.qshgm.lbci.net/. A user-friendly "'browse" option allows to explore the data including the annotated and extended entries. In the "browse" option, a query box is provided in which the user can enter the query on the basis of "All", "Synthases" or "Receptors" for the browsing of entries. By "Synthases", one can query entries according to nine QS languages: AHLs, CAI-1, Dialkylresorcinols, Photopyrones, DSFs, HAQs, AIPs, Indole, and AI-2. As an example, we have illustrated part of browsing results for AHLs language in Supplementary Figure 2, and the output displays information of the entries, fielded by Entry, Genus, Species, Strain, Taxonomic identifier (TaxID), Protein annotations, conventional abbreviations of QS signals (Languages), and Link Address.

QSHGM also includes "Search" searching facilities for different entries. In the search option, a query box is provided in which the user can enter the query on the basis of "Microbes", "Synthases" or "Receptors" for the searching of entries. By "Microbes", one can query entries according to different options: Entry (e.g., J7JCP9), Name (e.g., *Pseudomonas aeruginosa*), or TaxID (e.g., 208964). The output displays information of the entries, fielded by Entry, Organism from WMH, TaxID from Uniprot (Uniprot), Proteome ID, substitute organism (Organism), substitute organism TaxID (Organism ID), all protein counts (All Proteins), entry counts for the strain (Counts), and Protein annotations. The "Synthases" is provided according to different options: Entry (e.g., J7JCP9) or Languages (e.g., AHLs). By "Receptors", one can search with different options: Entry (e.g., P25084) or Annotations (e.g., Histidine kinase). The output displays information of entries, fielded by Entry, Genus, Species, Strain, TaxID, Protein annotations, and Languages. Note that search type allows users to retrieve either an exact match or the match containing the query.

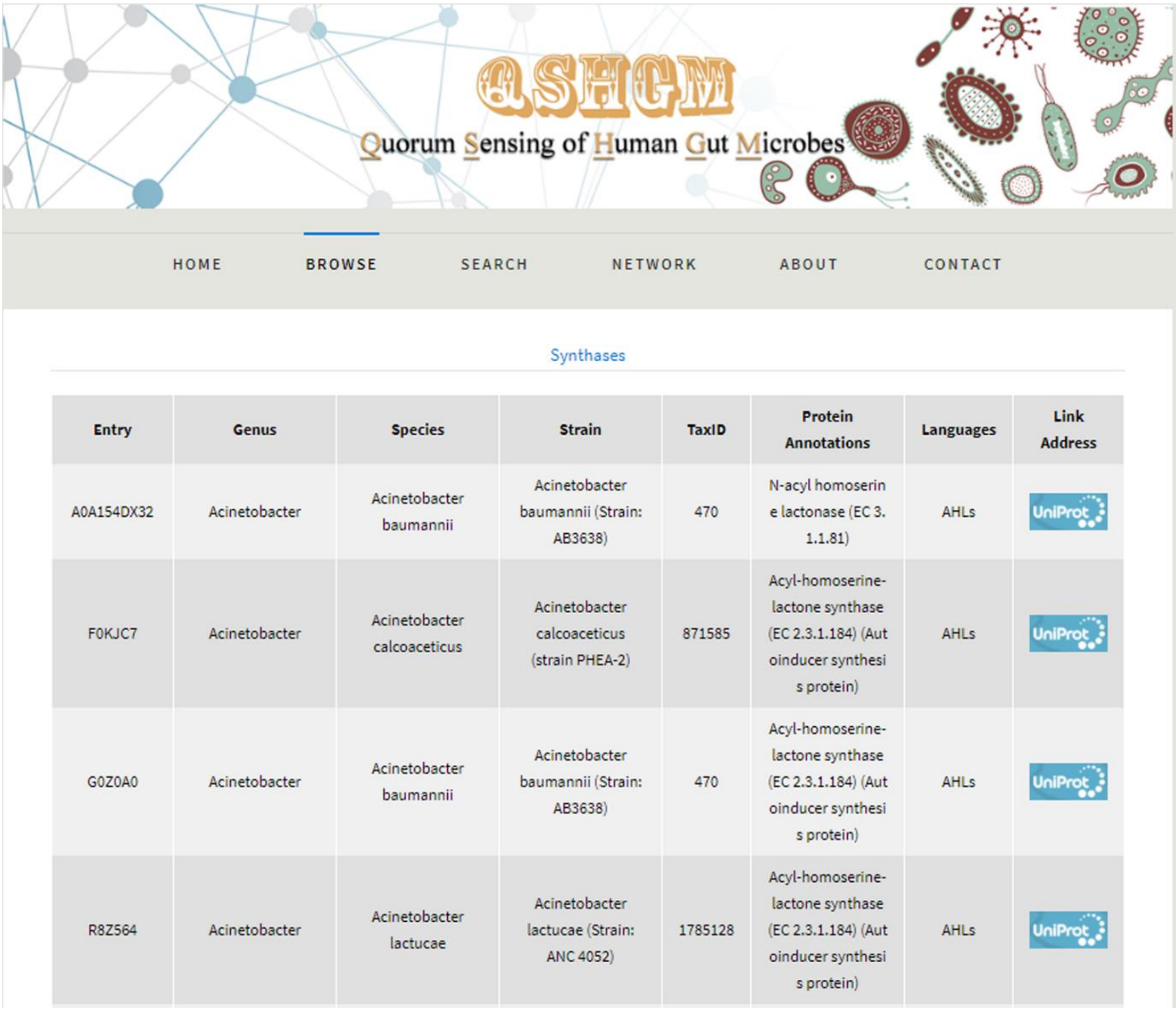

Entry	Genus	Species	Strain	TaxID	Protein Annotations	Languages	Link Address
A0A154DX32	Acinetobacter	Acinetobacter baumannii	Acinetobacter baumannii (Strain: AB3638)	470	N-acyl homoserine lactonase (EC 3.1.1.81)	AHLs	UniProt
F0KJC7	Acinetobacter	Acinetobacter calcoaceticus	Acinetobacter calcoaceticus (strain PHEA-2)	871585	Acyl-homoserine-lactone synthase (EC 2.3.1.184) (Autoinducer synthesis protein)	AHLs	UniProt
G0Z0A0	Acinetobacter	Acinetobacter baumannii	Acinetobacter baumannii (Strain: AB3638)	470	Acyl-homoserine-lactone synthase (EC 2.3.1.184) (Autoinducer synthesis protein)	AHLs	UniProt
R8Z564	Acinetobacter	Acinetobacter lactucae	Acinetobacter lactucae (Strain: ANC 4052)	1785128	Acyl-homoserine-lactone synthase (EC 2.3.1.184) (Autoinducer synthesis protein)	AHLs	UniProt

Supplementary Figure 3. Part of browsing results for AHLs language in QSHGM database.

4. QSCN illustration

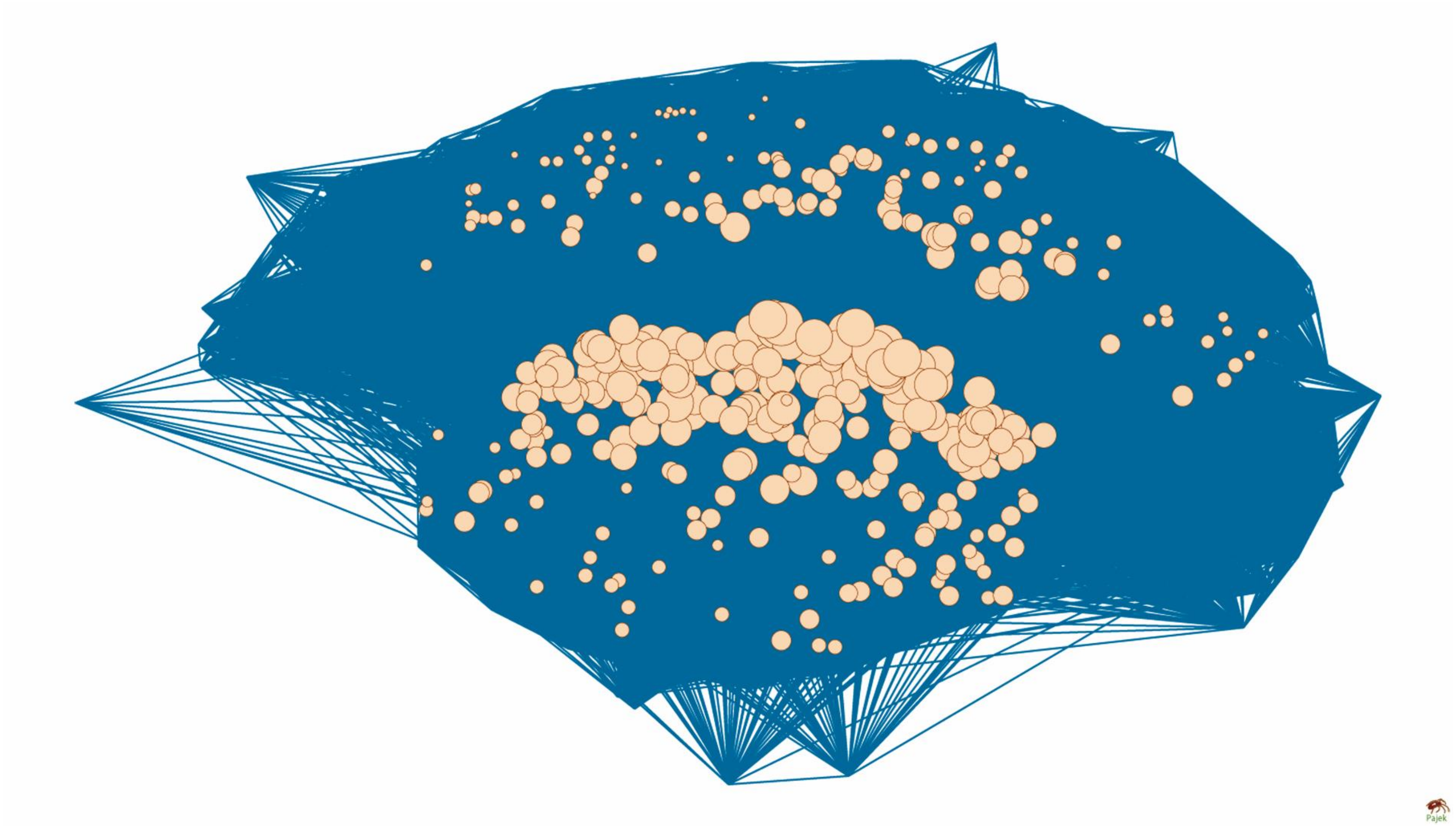

Supplementary Figure 4. Overview of the QS-based microbe-microbe network. Note that the network diagram was generated using Pajek [1].

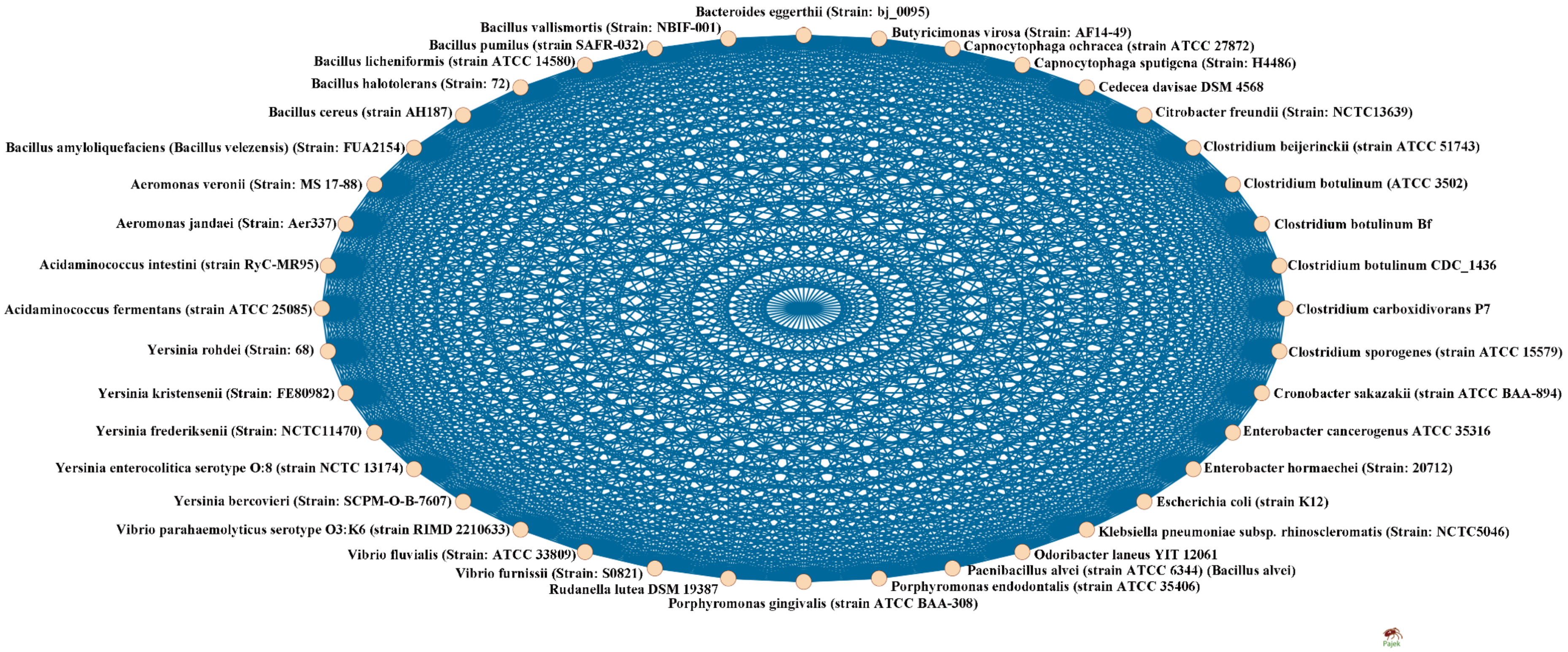

Supplementary Figure 5. A complete graph that links every node to every other node for the 40 core microbes.

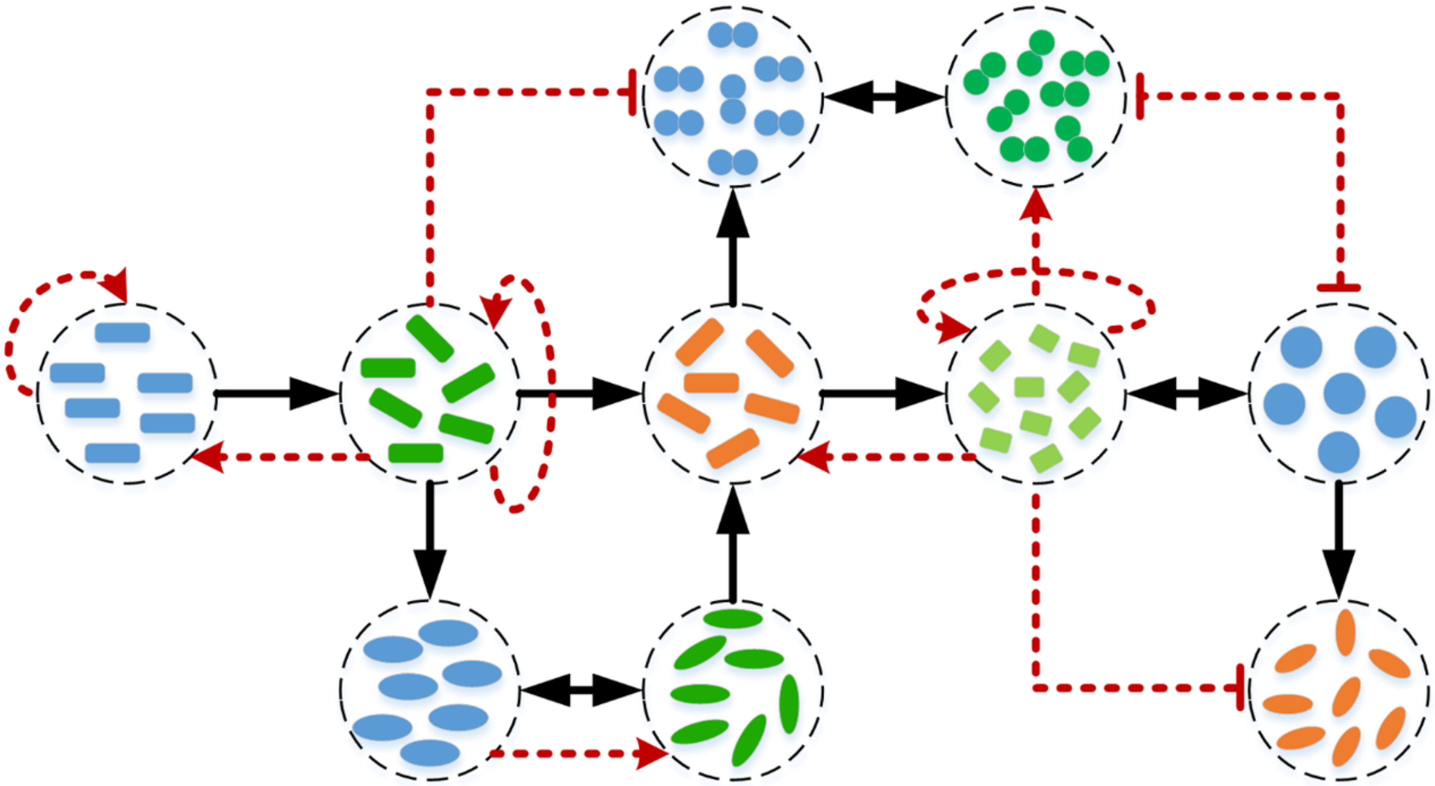

Supplementary Figure 6. Schematic diagram of the comprehensive microbial interaction framework including MIN and QSCN.

5. Case study for QSCN

Recently, some studies have described the development of alternative models of a simplified human microbiota consortium (SIHUMI), composed of 7-12 well-characterized and sequenced human-derived enteric bacteria, showing stable microbial and metabolomic characterization[2, 3]. Taking the case from Venturelli et al.[3] (involving species BT, BO, BU, BV, BH, CA, CH, DP, EL, ER, FP, and PC) that was based on multiplexed 16S rRNA gene sequencing, we have conducted a qualitative analysis to illustrate the necessity of a combining metabolic interchange network (MIN) and a QSCN. Note that the clustering pattern for the MIN (Original Figure 5e, Supplementary Figure 7b) did not fully recapitulate their interaction relationships of the 12 microbes (Original Figure 2c, Supplementary Figure 7a). Compared with the MIN, the total interaction network of the SIHUMI model is significantly denser, which are not included in the MIN. Venturelli et al. pointed out that signaling molecules may "contribute to the observed ecological relationships" in addition to the exchange of metabolites.

According to the data from the QSHGM database, we can construct the bipartite QSCN for the SIHUMI model (Supplementary Figure 7c). One can find that ten members of the community can communicate based on the AI-2 language (Supplementary Figure 7c), thus leading to a potentially dense QSCN. Note that there is no communication between BT and ER via a QS signal, which is in line with the result of the total interaction network (Supplementary Figure 7a). More importantly, the QSCN we constructed can offer a plausible QS-mediated mechanism for some specific links that their MIN could not explain:

(i) The prominent links between EL (rather isolated in the MIN) and other strains.

(ii) Links between CA (as another strain not well-connected in the MIN) and other strains.

(iii) Two further links, namely CH-DP and ER-BH.

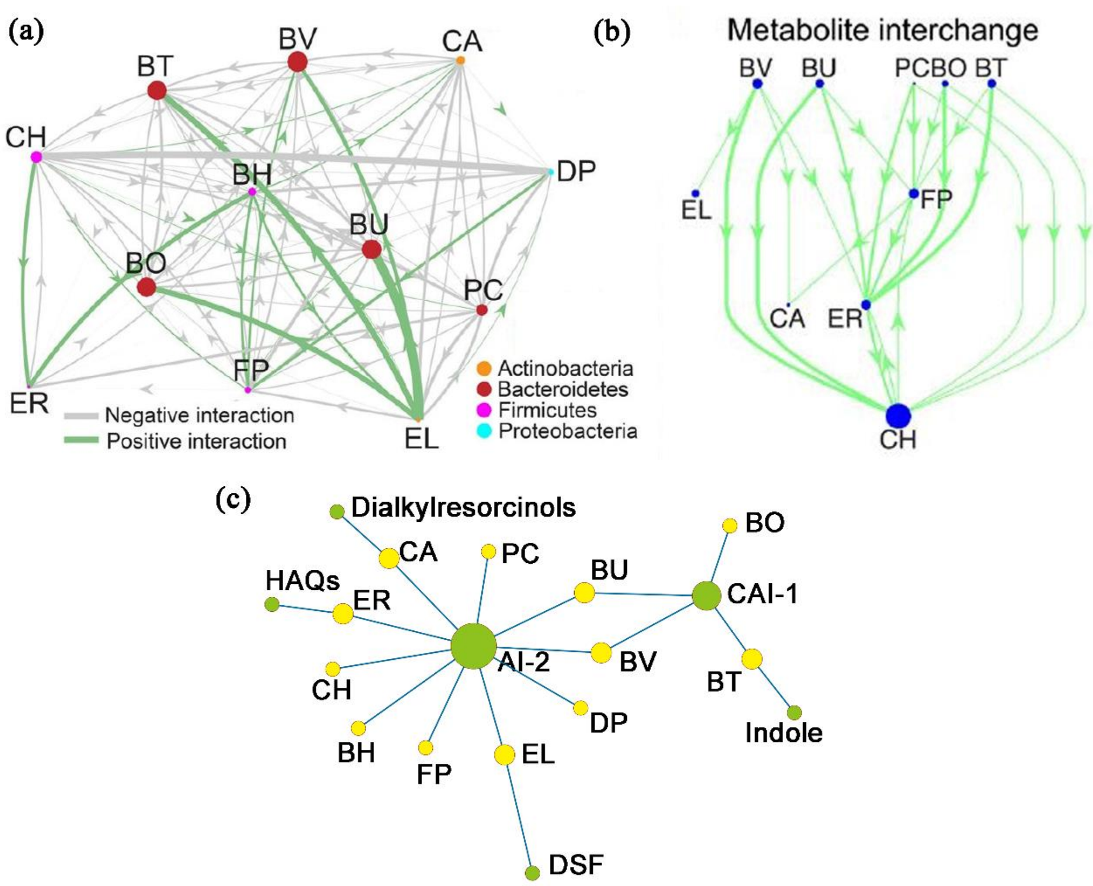

Supplementary Figure 7. Diverse networks for a SIHUMI model. (a) Inferred inter-species interaction for the gLV model trained on the time-resolved experiments from the original Figure 2c of Venturelli OS et al. [3]; (b) Predicted metabolite interchange network representing metabolites that were secreted or utilized by distinct organisms from the original Figure 5e of Venturelli OS et al. [3]; (c) Bipartite QSCN for the SIHUMI model based on QSHGM.

6. QS and TCS entries collection in the dataset III

We recognize the possible confusion around the two concepts of QS and Two-component signal transduction systems (TCSs) in this work. TCSs play an important role in microbial communications, which have a certain overlap with QS [4], but it is difficult to separate the two clearly. Generally, various QS systems can be roughly divided into three types: (i) acylated homoserine lactones (AHLs) and other autoinducers received by LuxR-type receptors utilized by Gram-negative bacteria; (ii) auto-inducing peptides (AIPs) and other autoinducers sensed by two-component systems utilized by Gram-positive bacteria; and (iii) autoinducer 2 (AI-2) and indole for interspecies communication of microbial communities [5]. Here, we have listed functions of the 213 QS entries collected into Dataset I (more details in Supplementary Data 1) in Supplementary Figure 6 to indicate the overlap of QS and TCS, which shows that TCSs form an important part of QS entries.

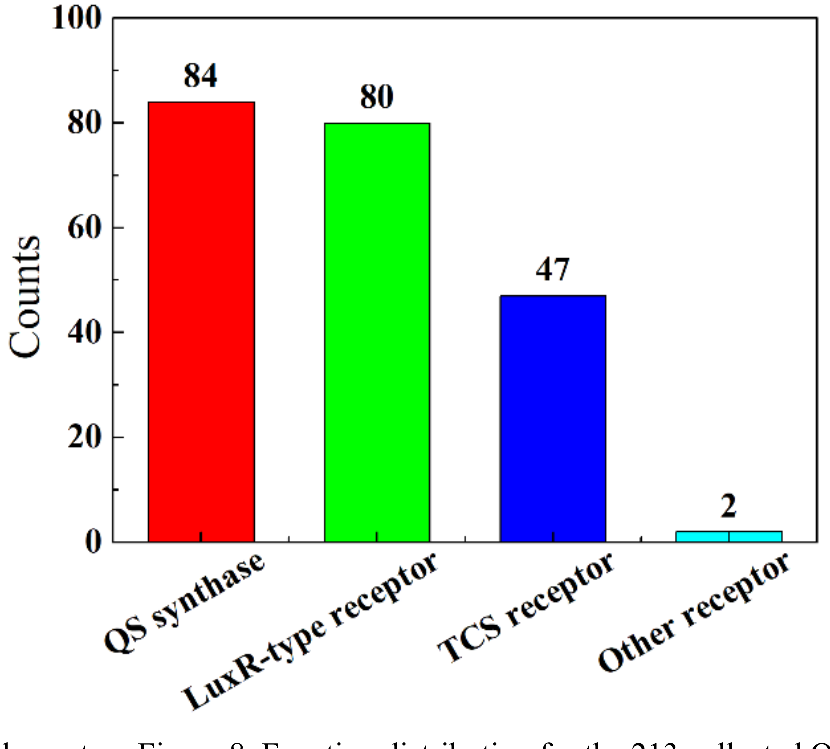

Supplementary Figure 8. Function distribution for the 213 collected QS entries.

While there is strong evidence from the 213 entries mentioned above that many TCS entries possess QS functionality, we agree that not all of them would do so, which would apply to a portion of the TCS entries collected into our Dataset III which was

built with the intention of collecting as many potentially QS-relevant entries as we can. On the other hand, we would like to point out that the entries in Dataset III (QS&TCS) was subsequently used as positive samples to train the classifiers; the predicted positive entries by applying the trained classifiers to the Dataset VII (resulting from Local BLASTP) were eventually checked manually to confirm QS functions. Out of 9253 entries (Dataset VII), 7184 were confirmed to be QS relevant, which represents a very high percentage. Therefore, although it is difficult to say exactly what is the proportion of the TCS entries collected in our final database that are not QS relevant, we anticipate that the proportion is likely to be moderate. Nevertheless, there is a need to make an explicit statement about the existence of such proportion so that the users are warned of encountering none-QS TCS entries, even though these entries would still be relevant to inter-cellular communication.

7. Potential applications of QSHGM and QSCN

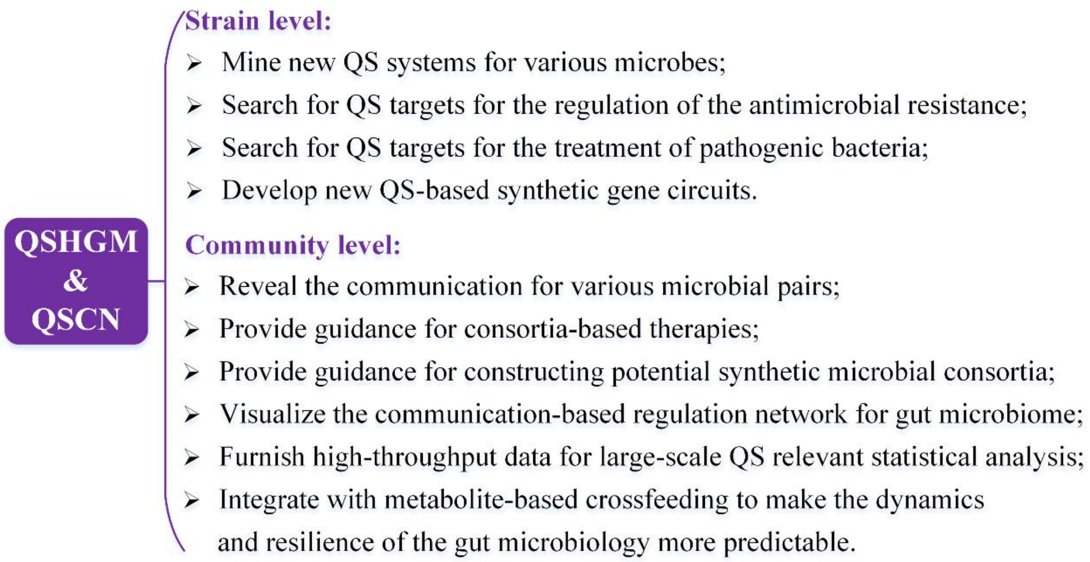

Supplementary Figure 9. Summarization for the potential applications of QSHGM and QSCN at strain and community level.

8. Tables for the corresponding content

Supplementary Table 1. The details for the datasets listed in the Figure 1.

Dataset	Input	Output	Suppl. Table
I	Reported entries from Sigmol and Quorumpeps database	**213** reported QS entries	1
II	Proteomes of human gut microbes from UniProt	Proteomes of **818** gut microbes from VMH.	https://pan.baidu.com/s/1o46nn1b7L5nvCqgpwW7Zlw **Password**: tfnx
III	Collected QS and TCS entries from Dataset II	Positive samples (**21,383** entries)	2
IV	Remove QS and TCS entries in cluster rules from typical strains in Dataset II	Negative samples (**22,780** entries)	3
V	Results of the Local BLASTP	Results of local BLASTP with $E \leq 10^{-5}$ (**14,573** entries)	4
VI	Overlaps of entries in Dataset III and V	**5,320** reported entries	5
VII	Entries by excluding Dataset VI for Dataset V	**9,253** entries to be classified	6
VIII	Union of uncharacterized proteins from the positives of RF, SVM, KNN, or DNN classifiers	**534** un-annotated entries to be mined	7
IX	The extended QS entries obtained by the union of four classifiers	**7,184** extended entries	8
Output S3	Proteins without QS functions	**438** false positives	9
X	The total entries from the reported and extended QS/TCS entries	**28,567** redundancy removal entries	10

Supplementary Table 2. Details of the selected nine types of QS languages

Type	Languages	Acronym	Synthase	Reported microbes	Refer.
Intra-species	Acyl-homoserine lactones	AHLs	Diverse I proteins (e.g., LuxI)	Most of Gram-negative bacteria (e.g., *Vibrio fischeri*)	6
	Diffusible signal factors	DSFs	RpfF	*Xanthomonas campestris*	7
	4-hydroxy-2-alkylquinoline	HAQs	PqsA	*Pseudomonas aeruginosa*	8
	Cholera autoinducer 1	CAI-1	CqsA	*Vibrio spp.*	9
	Dialkylresorcinols	DARs	DarB	*Photorhabdus asymbiotica*	10
	Photopyrones	-	PpyS	*Photorhabdus luminescens*	11
	Auto-inducing peptides	AIPs	Synthases of signal peptides (e.g., NisA)	Most of Gram-positive bacteria (e.g., *Lactococcus lactis*)	12
Inter-species	Indole	-	TnaA	Some microbes (e.g., *Escherichia coli*)	13
	Autoinducer 2	AI-2	LuxS	Most of microbes (e.g., *Vibrio harveyi*)	14

Supplementary Table 3. Hypotheses and future improvements for the QSCN

Items	Hypotheses	Future improvements for QSCN
Microbe	Human gut microbiome consists of 818 microbes from VMH database.	Enlarge the number and range of gut microbes.
Language	There are nine types of QS languages.	Develop the QSCN for more AIPs and novel QS languages.
TCS	TCS entries possess QS functionality.	Figure out the differences and connections between QS and TCS.
Cheating	All producers are also receivers to the same QS language.	Construct a directed and more accurate QSCN differentiating QS languages producing and receiving.
QS Crosstalk	Microbes that speak the same type of languages can communicate with each other.	Quantify the intensity of QS crosstalk for the same type of QS languages to develop a weighted QSCN.

Supplementary Table 4. Key nodes with high degree and betweenness centrality (BC)

Strains		Degree	BC
Acidaminococcus fermentans (strain ATCC 25085)	Firmicutes	712	0.00225
Acidaminococcus intestini (strain RyC-MR95)	Firmicutes	749	0.002698
Aeromonas jandaei (Strain: Aer337)	Proteobacteria	699	0.002438
Aeromonas veronii (Strain: MS 17-88)	Proteobacteria	699	0.002438
Bacillus amyloliquefaciens (Bacillus velezensis) (Strain: FUA2154)	Firmicutes	712	0.00225
Bacillus cereus (strain AH187)	Firmicutes	749	0.002698
Bacillus halotolerans (Strain: 72)	Firmicutes	712	0.00225
Bacillus licheniformis (strain ATCC 14580)	Firmicutes	749	0.002698
Bacillus pumilus (strain SAFR-032)	Firmicutes	749	0.002698
Bacillus vallismortis (Strain: NBIF-001)	Firmicutes	712	0.00225
Bacteroides eggerthii (Strain: bj_0095)	Bacteroidetes	702	0.002624
Butyricimonas virosa (Strain: AF14-49)	Bacteroidetes	723	0.002231
Capnocytophaga ochracea (strain ATCC 27872)	Bacteroidetes	746	0.003047
Capnocytophaga sputigena (Strain: H4486)	Bacteroidetes	723	0.002231
Cedecea davisae DSM 4568	Proteobacteria	714	0.002073
Citrobacter freundii (Strain: NCTC13639)	Proteobacteria	720	0.003004
Clostridium beijerinckii (strain ATCC 51743)	Firmicutes	711	0.00226
Clostridium botulinum (ATCC 3502)	Firmicutes	712	0.00225
Clostridium botulinum Bf	Firmicutes	711	0.002616
Clostridium botulinum CDC_1436	Firmicutes	712	0.00225
Clostridium carboxidivorans P7	Firmicutes	712	0.00225
Clostridium sporogenes (strain ATCC 15579)	Firmicutes	736	0.003093
Cronobacter sakazakii (strain ATCC BAA-894)	Proteobacteria	714	0.002073
Enterobacter cancerogenus ATCC 35316	Proteobacteria	714	0.002073
Enterobacter hormaechei (Strain: 20712)	Proteobacteria	736	0.002842
Escherichia coli (strain K12)	Proteobacteria	702	0.002624
Klebsiella pneumoniae subsp. rhinoscleromatis (Strain: NCTC5046)	Proteobacteria	736	0.002842
Odoribacter laneus YIT 12061	Bacteroidetes	748	0.003068
Paenibacillus alvei (strain ATCC 6344) (Bacillus alvei)	Firmicutes	771	0.003565
Porphyromonas endodontalis (strain ATCC 35406)	Bacteroidetes	723	0.002231
Porphyromonas gingivalis (strain ATCC BAA-308)	Bacteroidetes	723	0.002231
Rudanella lutea DSM 19387	Bacteroidetes	749	0.002698
Vibrio fluvialis (Strain: ATCC 33809)	Proteobacteria	751	0.003554
Vibrio furnissii (Strain: S0821)	Proteobacteria	730	0.002707
Vibrio parahaemolyticus serotype O3:K6 (strain RIMD 2210633)	Proteobacteria	730	0.002707
Yersinia bercovieri (Strain: SCPM-O-B-7607)	Proteobacteria	716	0.002234
Yersinia enterocolitica serotype O:8 (strain NCTC 13174)	Proteobacteria	730	0.002707
Yersinia frederiksenii (Strain: NCTC11470)	Proteobacteria	730	0.002707
Yersinia kristensenii (Strain: FE80982)	Proteobacteria	728	0.002542
Yersinia rohdei (Strain: 68)	Proteobacteria	730	0.002707

Supplementary Table 5. Design and optimization of synthetic microbial consortia based on QS

Time	QS system	Microbial community	Achieved function	Refs.
2002	*lux*	*E. coli*	Synchronizing genetic relaxation oscillators by intercell signaling	15
2004	*lux*	*E. coli*	Population control of an *E. coli* population by QS-regulated killing	16
2008	*lux*, *las*	*E. coli-E. coli*	Construct a *E. coli* predator–prey ecosystem	17
2009	*lux*, *las*	*E. coli-E. coli*	Spatial and temporal dynamics of a QS-regulated synthetic ecosystem	18
2010	*lux*	*E. coli*	Construct a synchronized QS-regulated genetic clocks	19
2012	*las*	*E. coli-E. coli*	Synthetic QS circuit to control consortial biofilm formation and dispersal	20
2014	*las*, *rhl*	*P. aeruginosa*	Combinatorial QS to resolve microbial social and physical environment	21
2015	*cin, rhl*	*E. coli-E. coli*	Investigate emergent genetic oscillations in a synthetic microbial consortium	22
2016	*lux*, *las*, *rpa*, *tra*	*E. coli-E. coli*	Investigate QS communication modules for microbial consortia	23
2017	*lux, rpa*	*S. typhimurium-S. typhimurium*	A stabilized microbial ecosystem by QS-based synchronized lysis circuits	24
2017	*esa*	*E. coli*	Construct a QS-based metabolic toggle switch for myo-inositol production	25
2018	*AIP* (nisin)	*L. lactis-L. lactis-L. lactis*	Designing microbial consortia with defined social interactions based on nisin	26
2018	*rhl, lux, tra, las, cin, rpa*	*E. coli-E. coli-E. coli-*	Engineer the coordinated system behavior in synthetic microbial consortia	27
2019	*lux*	*E. coli-E. coli-E. coli*	QS-based self-limiting synchronized lysis for microbial population	28
2019	*cin, rhl*	*E. coli-E. coli*	Long-range temporal coordination of gene expression in synthetic consortia	29
2020	*cin, rhl*	*E. coli-E. coli*	Majority sensing in synthetic microbial consortia	30
2020	*lux,irpa*	*E. coli-E. coli*	Construct inducible QS-regulated synthetic microbial communities	31
2020	*AIP*	*L. lactis-L. lactis*	Interaction variability shapes succession of synthetic microbial ecosystems	32
2021	Bacteriocin QS	*E. coli-E. coli-E. coli*	Design of synthetic consortia with QS-based amensal bacteriocin interaction	33
2021	*lux, las*	*E. coli-E. coli*	Combinational QS devices for dynamic control in cocultivation	34

Supplementary Table 6. Different characteristics of MINs and QSCNs

Items	MIN	QSCN
**Type**	Metabolite utilization network	Population-level signaling network
**Basis**	Metabolism of various substances or intermediates	Quorum sensing regulation systems
**Nodes**	Strains	Microbial communities
**Edges**	Metabolic interactions	Autoinducer-based regulations
**Molecules**	Various common metabolites (such as amino acid)	Diverse specific autoinducers (such as AHLs and AI-2)
**Proteins**	Enzymes are the majority (such as enzymes in glycolysis)	Specific QS receptors (such as LuxR, LsrB, SdiA)
**Interactions**	Catalytic reactions of different substrates or intermediates	Binding of autoinduces and receptors
**Analytical tools**	Reconstruction of metabolic networks (such as FBA) and other constraint-based modelling	Modelling based on corresponding QS mechanisms
**Functional nature**	Conservation of substance	Genetic circuits regulation
**Applications**	Optimization of metabolic engineering; microbial ecology analysis	Design of synthetic microbial consortia; microbial ecology analysis

References

1. Batagelj V, Mrvar A. Pajek-program for large network analysis. 11 (1998).
2. Colosimo DA*, et al.* Mapping interactions of microbial metabolites with human g-protein-coupled receptors. *Cell. Host. Microbe.* **26**, 273-282 e277 (2019).
3. Venturelli OS*, et al.* Deciphering microbial interactions in synthetic human gut microbiome communities. *Mol. Syst. Biol.* **14**, e8157 (2018).
4. Wang S, Payne GF, Bentley WE. Quorum sensing communication: Molecularly connecting cells, their neighbors, and even devices. *Annu. Rev. Chem. Biomol. Eng.* **11**, 447-468 (2020).
5. Wu S, Liu J, Liu C, Yang A, Qiao J. Quorum sensing for population-level control of bacteria and potential therapeutic applications. *Cell. Mol. Life Sci.* **77**, 1319-1343 (2020).
6. Fuqua WC, Winans SC, Greenberg EP. Quorum sensing in bacteria: The luxr-luxi family of cell density-responsive transcriptional regulators. *J. Bacteriol.* **176**, 269-275 (1994).
7. Deng YY, Wu JE, Tao F, Zhang LH. Listening to a new language: Dsf-based quorum sensing in gram-negative bacteria. *Chem. Rev.* **111**, 160-173 (2011).
8. Déziel E*, et al.* Analysis of pseudomonas aeruginosa 4-hydroxy-2-alkylquinolines (haqs) reveals a role for 4-hydroxy-2-heptylquinoline in cell-to-cell communication. *Proc Natl Acad Sci U S A* **101**, 1339-1344 (2004).
9. Higgins DA, Pomianek ME, Kraml CM, Taylor RK, Semmelhack MF, Bassler BL. The major vibrio cholerae autoinducer and its role in virulence factor production. *Nature* **450**, 883-886 (2007).
10. Brameyer S, Kresovic D, Bode HB, Heermann R. Dialkylresorcinols as bacterial signaling molecules. *Proceedings of the National Academy of Sciences* **112**, 572-577 (2015).
11. Brachmann AO*, et al.* Pyrones as bacterial signaling molecules. *Nat. Chem. Biol.* **9**, 573-578 (2013).
12. Monnet V, Juillard V, Gardan R. Peptide conversations in gram-positive bacteria. *Crit. Rev. Microbiol.* **42**, 339-351 (2014).
13. Wang D, Ding X, Rather PN. Indole can act as an extracellular signal in escherichia coli. *J. Bacteriol.* **183**, 4210-4216 (2001).
14. Chen X*, et al.* Structural identification of a bacterial quorum-sensing signal containing boron. *Nature* **415**, 545-549 (2002).
15. David M, Nancy K, Jeff H, J J C. Synchronizing genetic relaxation oscillators by intercell signaling. *Proc. Natl Acad. Sci. USA* **99**, 679-684 (2002).
16. You L, Cox RS, 3rd, Weiss R, Arnold FH. Programmed population control by cell-cell communication and regulated killing. *Nature* **428**, 868-871 (2004).
17. Balagaddé FK*, et al.* A synthetic escherichia coli predator-prey ecosystem. *Mol. Syst. Biol.* **4**, 187 (2008).
18. Song H, Payne S, Gray M, You L. Spatiotemporal modulation of biodiversity in

a synthetic chemical-mediated ecosystem. *Nat. Chem. Biol.* **5**, 929-935 (2009).
19. Danino T, Mondragon-Palomino O, Tsimring L, Hasty J. A synchronized quorum of genetic clocks. *Nature* **463**, 326-330 (2010).
20. Hong SH, Hegde M, Kim J, Wang X, Jayaraman A, Wood TK. Synthetic quorum-sensing circuit to control consortial biofilm formation and dispersal in a microfluidic device. *Nat. Commun.* **3**, 613 (2012).
21. Cornforth DM*, et al.* Combinatorial quorum sensing allows bacteria to resolve their social and physical environment. *Proc Natl Acad Sci U S A* **111**, 4280-4284 (2014).
22. Chen Y, Kim JK, Hirning AJ, Josic K, Bennett MR. Synthetic biology. Emergent genetic oscillations in a synthetic microbial consortium. *Science* **349**, 986-989 (2015).
23. Scott SR, Hasty J. Quorum sensing communication modules for microbial consortia. *Acs. Synth. Biol.* **5**, 969-977 (2016).
24. Scott SR, Din MO, Bittihn P, Xiong L, Tsimring LS, Hasty J. A stabilized microbial ecosystem of self-limiting bacteria using synthetic quorum-regulated lysis. *Nat. Microbiol.* **2**, 17083 (2017).
25. Gupta A, Reizman IM, Reisch CR, Prather KL. Dynamic regulation of metabolic flux in engineered bacteria using a pathway-independent quorum-sensing circuit. *Nat. Biotechnol.* **35**, 273-279 (2017).
26. Kong W, Meldgin DR, Collins JJ, Lu T. Designing microbial consortia with defined social interactions. *Nat. Chem. Biol.* **14**, 821-829 (2018).
27. Kylilis N, Tuza ZA, Stan GB, Polizzi KM. Tools for engineering coordinated system behaviour in synthetic microbial consortia. *Nat. Commun.* **9**, 2677 (2018).
28. Liao MJ, Din MO, Tsimring L, Hasty J. Rock-paper-scissors: Engineered population dynamics increase genetic stability. *Science* **365**, 1045-1049 (2019).
29. Kim JK, Chen Y, Hirning AJ, Alnahhas RN, Josic K, Bennett MR. Long-range temporal coordination of gene expression in synthetic microbial consortia. *Nat. Chem. Biol.* **15**, 1102-1109 (2019).
30. Alnahhas RN*, et al.* Majority sensing in synthetic microbial consortia. *Nat. Commun.* **11**, 3659 (2020).
31. Miano A, Liao MJ, Hasty J. Inducible cell-to-cell signaling for tunable dynamics in microbial communities. *Nat. Commun.* **11**, 1193 (2020).
32. Liu F*, et al.* Interaction variability shapes succession of synthetic microbial ecosystems. *Nat. Commun.* **11**, 309 (2020).
33. Karkaria BD, Fedorec AJH, Barnes CP. Automated design of synthetic microbial communities. *Nat. Commun.* **12**, 672 (2021).
34. Wu S*, et al.* Combinational quorum sensing devices for dynamic control in cross-feeding cocultivation. *Metab. Eng.* **67**, 186-197 (2021).
