# Supplementary material for: Machine learning aided construction of the quorum sensing communication network for human gut microbiota
Source: Nat Commun. 2022 Jun 2;13:3079. doi: 10.1038/s41467-022-30741-6 (PMC9163137; doi:10.1038/s41467-022-30741-6)
Supplement: Supplementary file 15 — Reporting Summary [file 41467_2022_30741_MOESM15_ESM.pdf]

nature portfolio

Corresponding author(s): NCOMMS-21-29458D

Last updated by author(s): May 7 2022

Reporting Summary

Nature Portfolio wishes to improve the reproducibility of the work that we publish. This form provides structure for consistency and transparency in reporting. For further information on Nature Portfolio policies, see our Editorial Policies and the Editorial Policy Checklist.

Statistics

For all statistical analyses, confirm that the following items are present in the figure legend, table legend, main text, or Methods section.

n/a	Confirmed	
☐	☒	The exact sample size (*n*) for each experimental group/condition, given as a discrete number and unit of measurement
☐	☒	A statement on whether measurements were taken from distinct samples or whether the same sample was measured repeatedly
☒	☐	The statistical test(s) used AND whether they are one- or two-sided<br>*Only common tests should be described solely by name; describe more complex techniques in the Methods section.*
☒	☐	A description of all covariates tested
☐	☒	A description of any assumptions or corrections, such as tests of normality and adjustment for multiple comparisons
☐	☒	A full description of the statistical parameters including central tendency (e.g. means) or other basic estimates (e.g. regression coefficient) AND variation (e.g. standard deviation) or associated estimates of uncertainty (e.g. confidence intervals)
☒	☐	For null hypothesis testing, the test statistic (e.g. *F*, *t*, *r*) with confidence intervals, effect sizes, degrees of freedom and *P* value noted<br>*Give P values as exact values whenever suitable.*
☒	☐	For Bayesian analysis, information on the choice of priors and Markov chain Monte Carlo settings
☐	☒	For hierarchical and complex designs, identification of the appropriate level for tests and full reporting of outcomes
☒	☐	Estimates of effect sizes (e.g. Cohen's *d*, Pearson's *r*), indicating how they were calculated

Our web collection on statistics for biologists contains articles on many of the points above.

Software and code

Policy information about availability of computer code

Data collection	We firstly obtained 213 recognized QS entries (Dataset I) from SigMol and Quorumpeps databases, and curated their corresponding amino acid sequences from the UniProt database. In parallel, we manually searched the 818 gut microbes from the VMH database (Dataset II) to collect reported QS entries. We have also provided computer readable tables in Supplementary Tables 1-13. We also used Python 3.7 to write and analyze the method and the collected data to construct ensemble classifiers. The codes have been provided in a GitHub repository at the following URL:https://github.com/guofei-tju/qshgm-code
Data analysis	We have used some reported softwares, including MEGA X, iTOL, BLASTP, EVenn, iFeature, MultiScheme package, and GridSearchCv, to conduct data analysis. We also used Python 3.7 to write and analyze the method and the collected data to construct ensemble classifiers. The codes have been provided in a GitHub repository at the following URL:https://github.com/guofei-tju/qshgm-code

For manuscripts utilizing custom algorithms or software that are central to the research but not yet described in published literature, software must be made available to editors and reviewers. We strongly encourage code deposition in a community repository (e.g. GitHub). See the Nature Portfolio guidelines for submitting code & software for further information.

Data

Policy information about availability of data

All manuscripts must include a data availability statement. This statement should provide the following information, where applicable:

- Accession codes, unique identifiers, or web links for publicly available datasets
- A description of any restrictions on data availability
- For clinical datasets or third party data, please ensure that the statement adheres to our policy

QSHGM, a database of 28,567 redundancy removal QS synthases (1,882) and receptors (26,685) entries for 818 gut microbes, which is freely available at: (http://

www.qshgm.lbci.net/). We have also provided computer readable tables in Supplementary Tables 1-13.

Field-specific reporting

Please select the one below that is the best fit for your research. If you are not sure, read the appropriate sections before making your selection.

☒ Life sciences ☐ Behavioural & social sciences ☐ Ecological, evolutionary & environmental sciences

For a reference copy of the document with all sections, see nature.com/documents/nr-reporting-summary-flat.pdf

Life sciences study design

All studies must disclose on these points even when the disclosure is negative.

Sample size	In the collecting module, we firstly obtained 213 recognized QS entries (Dataset I) from SigMol and Quorumpeps databases, and curated their corresponding amino acid sequences from the UniProt database. In this work, we started by manually searching the 818 gut microbes from the VMH database (Dataset II) to collect reported both QS and TCS (QS&TCS) entries which are termed "positive samples" (Dataset III, 21,383 entries). The negative samples (Dataset IV, 22,780 entries) were then obtained by removing QS&TCS entries from typical proteomes in Dataset II, such as Escherichia coli and Pseudomonas aeruginosa (more details in Method section) that conform to QS cluster rules. In the expanding module, we obtained an extended dataset (Dataset V, 14,573 entries) from the results of the local BLASTP on the Dataset I and II with the criteria of the E value being smaller than 0.00001. After excluding from Dataset V those which were already collected as the reported QS&TCS entries in dataset III (Dataset VI, 5,320 entries), the remaining entries (Dataset VII, 9,253 entries) were then classified by the four ML-based classifiers. The output of these classifiers was further processed in the mining module, where the union of the four positives predicted by the four classifiers were divided into uncharacterized positives (Dataset VIII, 534 entries) and annotated positives. Furthermore, we conducted the function analysis by checking their specific annotations, sequence similarity, and domains (see more details in Supplementary Table 11) for the annotated/re-annotated union of positives to decide whether the entry has QS function (true positives, Dataset IX, 7,184 entries) or not (false positives, Output S3, 438 entries), if so, whether it is a QS synthase or a QS receptor. Finally, the extended QS entries and the reported QS&TCS entries were combined together to form the QSHGM (Dataset X, 28,567 entries) database (http://www.qshgm.lbci.net/).
Data exclusions	No data were excluded
Replication	Since we used previously published data, and did not perform experiments at 3 biological replicates. We calculated the frequency of each amino acid type in each entry sequence as the protein features, and we conducted 5-fold cross validation to train classifiers using the positive (Dataset III) and negative samples (Dataset IV), where the average accuracy, prediction, recall, and F1 score (more details were listed in method section) were applied to evaluate their performances. On this basis, we carried out relevant analysis on the obtained data.
Randomization	Not relevant to our computational study since we did not have any experimental groups
Blinding	Not relevant since we did not assign any experimental groups

Reporting for specific materials, systems and methods

We require information from authors about some types of materials, experimental systems and methods used in many studies. Here, indicate whether each material, system or method listed is relevant to your study. If you are not sure if a list item applies to your research, read the appropriate section before selecting a response.

Materials & experimental systems

n/a	Involved in the study	
☒	☐	Antibodies
☒	☐	Eukaryotic cell lines
☒	☐	Palaeontology and archaeology
☒	☐	Animals and other organisms
☒	☐	Human research participants
☒	☐	Clinical data
☒	☐	Dual use research of concern

Methods

n/a	Involved in the study	
☒	☐	ChIP-seq
☒	☐	Flow cytometry
☒	☐	MRI-based neuroimaging
