# Supplementary material for: Machine learning aided construction of the quorum sensing communication network for human gut microbiota
Source: Nat Commun. 2022 Jun 2;13:3079. doi: 10.1038/s41467-022-30741-6 (PMC9163137; doi:10.1038/s41467-022-30741-6)
Supplement: Supplementary file 17 — Description of Additional Supplementary Files [file 41467_2022_30741_MOESM17_ESM.pdf]

Title: Supplementary Data 1.
Description: Reported QS entries from Sigmol and Quorumpeps.

Title: Supplementary Data 2.
Description: Positive samples for the collected QS entries.

Title: Supplementary Data 3.
Description: Negative samples collection.

Title: Supplementary Data 4.
Description: Results of local BLASTP with E ≤ 10-5 (Dataset V).

Title: Supplementary Data 5.
Description: Overlaps of QS entries in dataset III and V (Dataset VI).

Title: Supplementary Data 6.
Description: Protein dataset excluded dataset VI for dataset V (VII).

Title: Supplementary Data 7.
Description: Uncharacterized proteins from four classifiers (VIII).

Title: Supplementary Data 8.
Description: Extended entries obtained by four classifiers (IX).

Title: Supplementary Data 9.
Description: False positives obtained by four classifiers (S3).

Title: Supplementary Data 10.
Description: Data for QSHGM database (X).

Title: Supplementary Data 11.
Description: Function analysis for positive entries.

Title: Supplementary Data 12.
Description: Overlaps for AP and UP.

Title: Supplementary Data 13.
Description: QS Communication network data for pajek.
